# Supplementary material for: Controlling Unconventional Secretion for Production of Heterologous Proteins in Ustilago maydis through Transcriptional Regulation and Chemical Inhibition of the Kinase Don3
Source: J Fungi (Basel). 2021 Mar 3;7(3):179. doi: 10.3390/jof7030179 (PMC7999842; doi:10.3390/jof7030179)
Supplement: Supplementary file 1 [file jof-07-00179-s001.pdf]

Journal of
Fungi

MDPI

Supplementary Material

Controlling Unconventional Secretion for Production of Heterologous Proteins in *Ustilago maydis* Through Transcriptional Regulation and Chemical Inhibition of the Kinase Don3

Kai P. Hussnaetter, Magnus Philipp, Kira Müntjes, Michael Feldbrügge and Kerstin Schipper

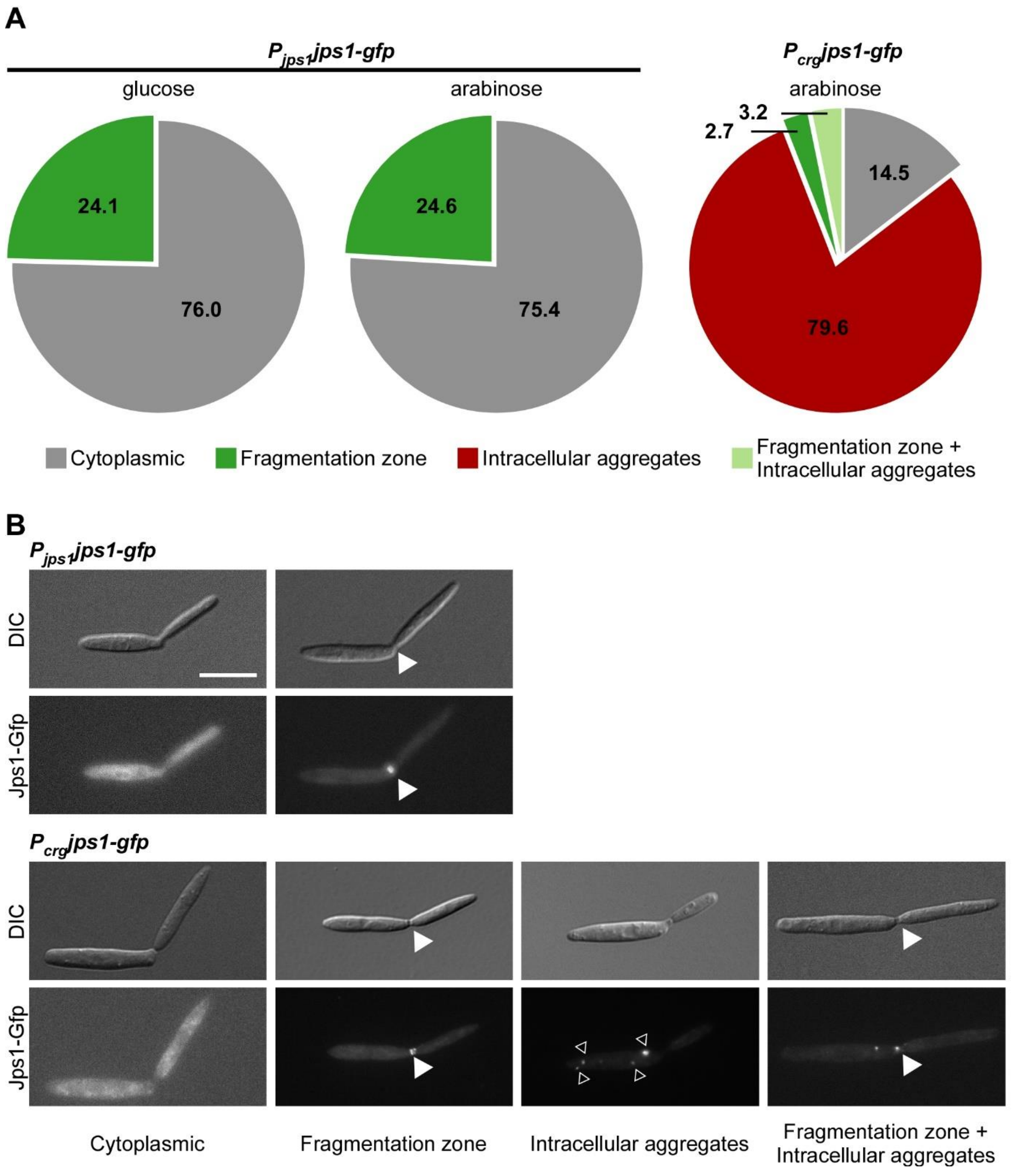

Figure S1. Influence of transcriptional regulation by an arabinose-inducible promoter on Jps1-Gfp localization. Localization of Jps1-Gfp in the control strain AB33jps1Δ/P_{jps1}jps1-gfp (*P_{jps1}jps1-gfp*) in which *jps1-gfp* expression is controlled by the native promoter P_{jps1}, and the regulable strain AB33jps1Δ/P_{crg}jps1-eGfp/Gus-Cts1 in which *jps1-gfp* expression is regulated by arabinose inducible promoter P_{crg}. The control strain was grown in both glucose and arabinose to exclude medium effects. The regulable strain was only grown in arabinose for induction of *jps1-gfp* expression. Yeast-like growing cells in all stages of cytokinesis were analyzed. (**A**) Pie charts depict ratios of the different observed localization patterns of Gfp fluorescence: cytoplasmic, fragmentation zone intracellular aggregates, and fragmentation zone + intracellular aggregates. The experiment was conducted in one biological replicate with a total of n=186 cells analyzed (*P_{crg}jps1-gfp*, control strains: 130 cells; *P_{jps1}:jps1-gfp* after growth in glucose; 79 cells *P_{jps1}jps1-gfp* after growth in arabinose). Percentage of cells in different categories is shown in pie charts. (**B**) Micrographs depicting cells of both strains grown in CM-arabinose. White arrowheads indicate the expected localization of Jps1-Gfp in the fragmentation zone [30], open white arrowheads depict intracellular aggregates. DIC, differential interference contrast; Jps1-Gfp, Gfp signal. Scale bar, 10 μm.

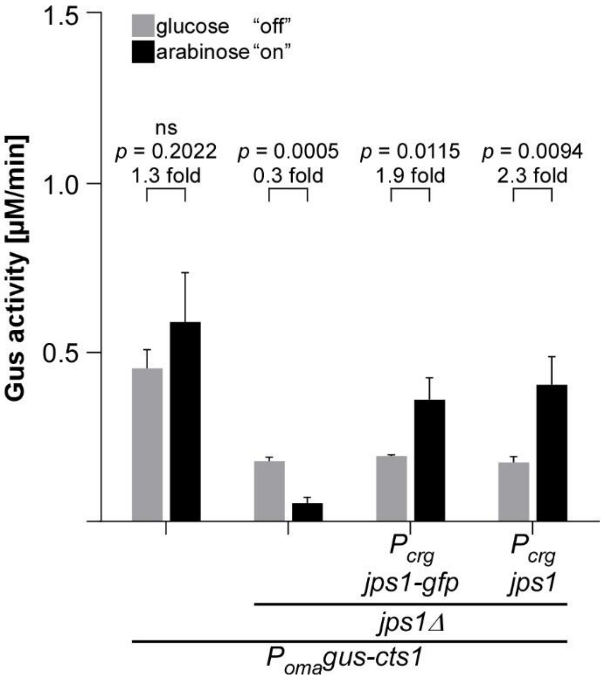

Figure S2. Comparative absolute Gus activity for the assay depicted in Fig. 2C. Enzyme activity is shown in μM/min, for Figure 2C, average values of control strain AB33 Gus-Cts1 (*P_{oma}gus-cts1*) were set to 1 and used as reference for other values. No significant difference for the reference strain grown in glucose or arabinose medium was detectable (*p* = 0.2022). The diagram represents results of three biological replicates. Error bars depict standard deviation. Fold change of cultures and *p*-values of Student's unpaired *t*-test are shown. Definition of statistical significance: *p*-value < 0.05.

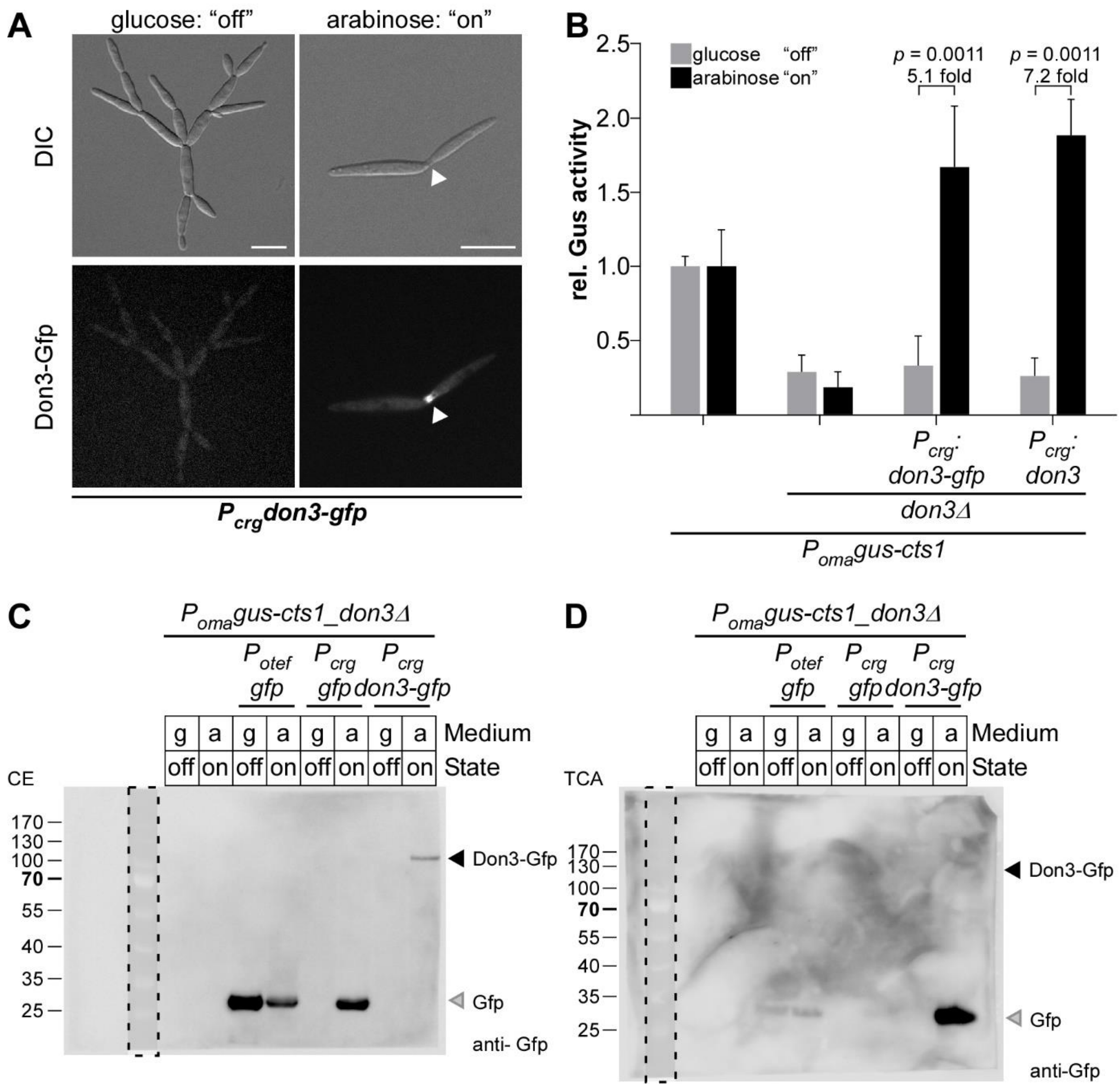

Figure S3. Additional data on transcriptional regulation of unconventional secretion via kinase Don3. (**A**) Micrographs of yeast-like growing cells in the "on" and "off" stage mediated by glucose and arabinose in the medium, respectively. Arrowheads depict the Gfp signal at the mother-daughter cell boundary. DIC, differential interference contrast. Scale bars, 10 µm. (**B**) Gus activity in culture supernatants of indicated AB33 Gus-Cts1 derivatives. Enzymatic activity was individually normalized to average values of positive controls secreting Gus-Cts1 constitutively, which were grown in glucose and arabinose containing cultures. Values of positive controls in the two media do not differ significantly ($p = 0.4820$; Fig. S4). Strains containing regulated *don3* or *don3-gfp* versions show a strong induction of extracellular Gus activity after growth in arabinose containing medium. Error bars depict standard deviation. The diagram represents results of four biological replicates. Fold change of induced cultures and *p*-values of Student's unpaired *t*-test are shown. Definition of statistical significance: p-value < 0.05. Complete Western blots of selected signals shown in Fig. 3D. (**C**) Cell extracts (CE) and (**D**) TCA precipitated supernatants (TCA) of AB33don3Δ/Gus-Cts1 derivatives are shown. Cells were cultivated either in glucose (g) or in arabinose (a) containing medium, resulting in "off" or "on" state of unconventional secretion, respectively. Primary antibodies against Gfp were used to detect the respective proteins (anti-Gfp). Selected strains either express no *gfp* (negative control), *gfp* under the control of a constitutive promoter as positive control (P_{otef}*gfp*), *gfp* under the control of an inducible promoter (P_{crg}*gfp*), or *don3-gfp* under the control of the inducible *crg* promoter (P_{crg}*don3-gfp*), from left to right, respectively. Black arrowhead indicates Don3-Gfp signal (predicted size: 118 kDa), gray arrowheads depict the Gfp signal (predicted size: 27 kDa). Dashed area indicates digitization of protein marker with 70% opacity.

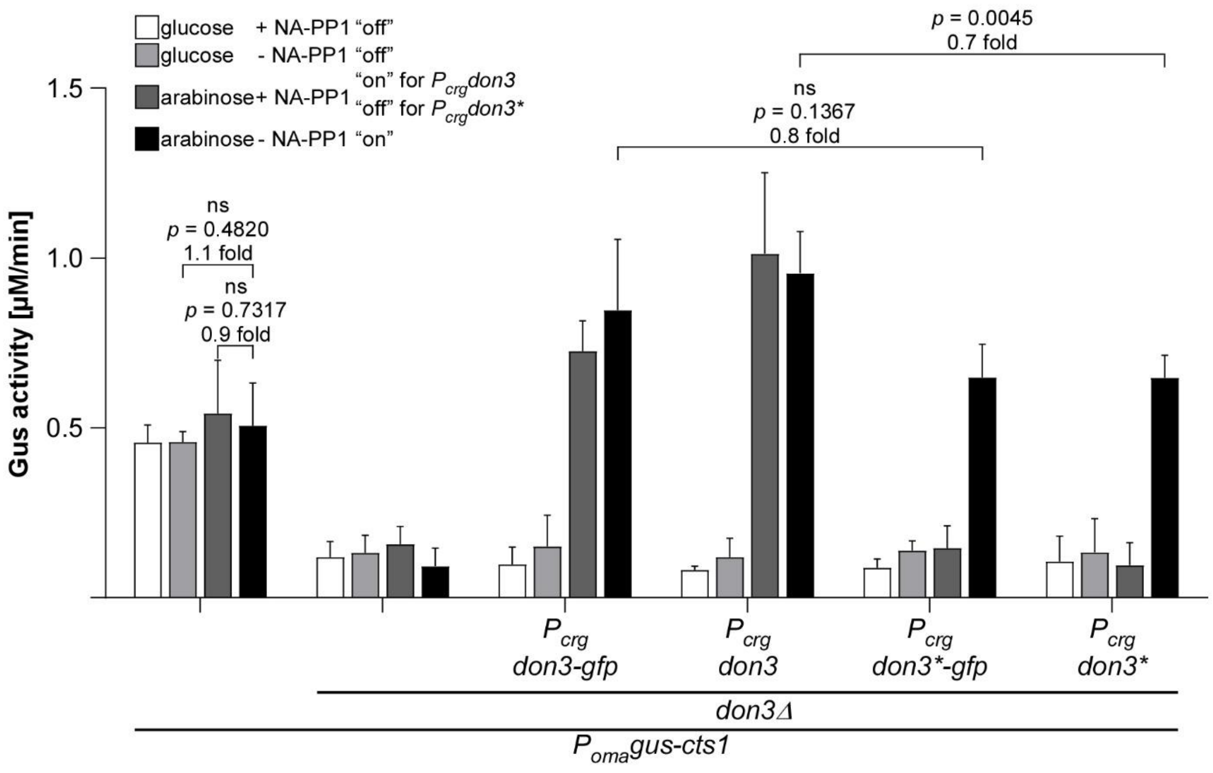

Figure S4. Comparative absolute Gus activity for the assays depicted in Figs. S3B and 4C. Extracellular Gus activity of all inducible Don3 strains and controls was determined in all four different media. Enzyme activity is shown in µM/min, for Figure 3C and 4C, average values of control strain AB33 Gus-Cts1 (*P_{oma}gus-cts1*) in respective media were set to 1 and used as reference for other values. No significant difference for reference strain grown in glucose or arabinose medium was detectable (P=0.4820, 0.7313). A 0.8-fold, yet not significant change was detected for AB33don3Δ/P_{crg}don3-gfp/Gus-Cts1 (*P_{crg}don3-gfp*) compared to AB33don3Δ/P_{crg}don3*-gfp/Gus-Cts1 (*P_{crg}don3*-gfp*), while a significant 0.7 fold change is detectable for AB33don3Δ/P_{crg}don3/Gus-Cts1 (*P_{crg}don3*) compared to AB33don3Δ/P_{crg}don3*/Gus-Cts1 (*P_{crg}don3**), suggesting a slightly reduced activity of the post-translational regulated system in comparison to the transcriptional regulated system. The diagram represents results of four biological replicates. Error bars depict standard deviation. Fold change of cultures and *p*-values of Student's unpaired *t*-test are shown. Definition of statistical significance: *p*-value < 0.05.

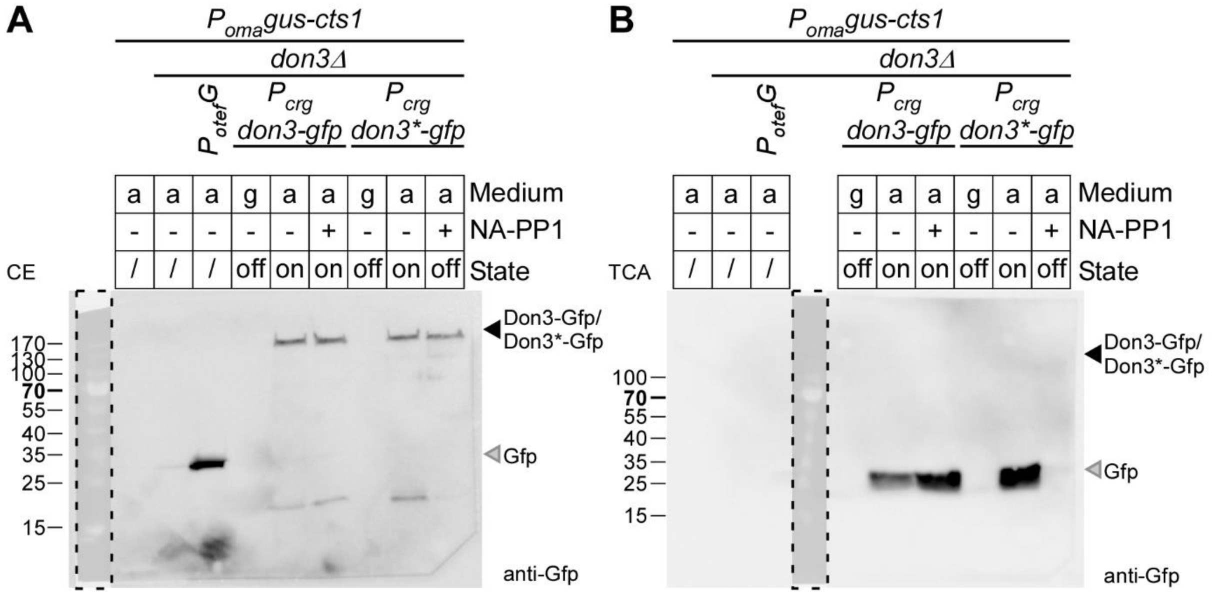

Figure S5. Complete Western blots of selected signals shown in Fig. 4D. (**A**) Cell extracts (CE) and (**B**) TCA precipitated supernatants (TCA) of AB33 Gus-Cts1 derivatives are shown. Cells were cultivated in medium containing either glucose (g) or arabinose (a), with (+) or without (-) ATP-analog NA-PP1, resulting in either "on" or "off" state of unconventional

secretion. Primary antibodies against Gfp were used to detect the respective proteins (anti-Gfp). Control strains were only cultivated in arabinose medium without NA-PP1 and express either no *gfp* (P_{oma}*gus-cts1* and P_{oma}*gus-cts1/don3Δ*) or *gfp* constitutively (P_{otef}*gfp*). Inducible secretion strains, expressing either *don3-gfp* or *don3*-gfp* cultivated in all three different media are shown on the right. Black arrowheads indicate Don3-Gfp signal (predicted size: 118 kDa), gray arrowheads indicate Gfp signal (predicted size: 27 kDa). Proteins run slightly higher than expected. This phenomenon was observed before [14]. Dashed area indicates digitization of protein marker with 70% opacity.

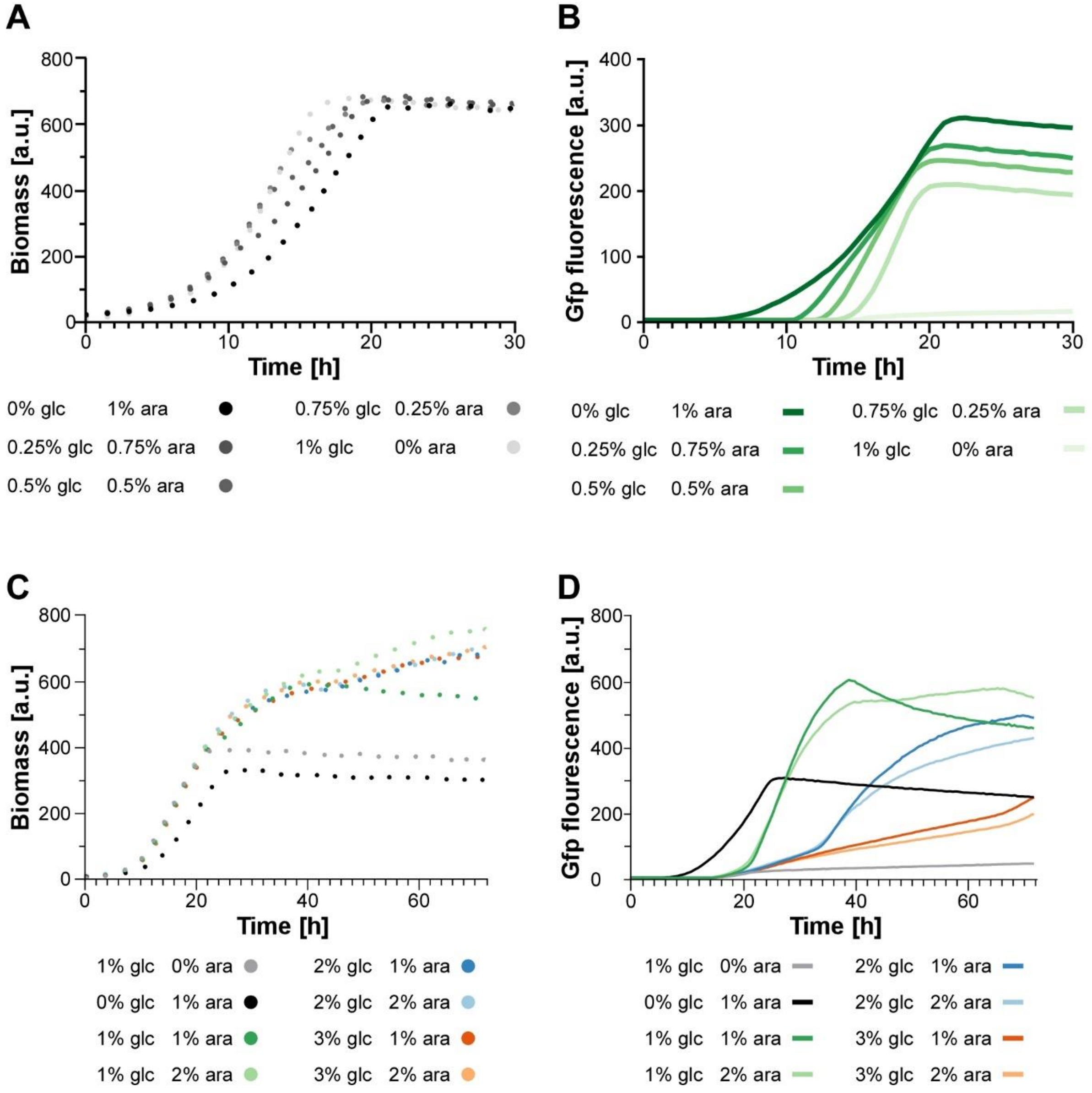

Figure S6. Additional data on establishment of an autoinduction process based on transcriptional regulation via carbon source switch. Strain AB33don3Δ/P_{crg}Gfp/Gus-Cts1 was used as a reporter for P_{crg} activity in buffered CM medium supplemented with different compositions of glucose and arabinose as carbon source. The two parameters fungal biomass and Gfp fluorescence were recorded online in a BioLector device. (**A**,**B**) P_{crg} activity in buffered CM medium supplemented with 1% total sugar in the indicated combinations. (A) Dotted lines represent fungal biomass, gain 25, (**B**) solid lines, Gfp fluorescence, gain 80. (**C**,**D**) Different glucose concentrations (1–3%) for initial growth were followed by either 1% or 2% arabinose for induction of unconventional secretion. (**C**) Dotted lines represent fungal biomass, gain 20, (**D**) solid lines, Gfp fluorescence, gain 80. (glc, glucose; ara, arabinose).

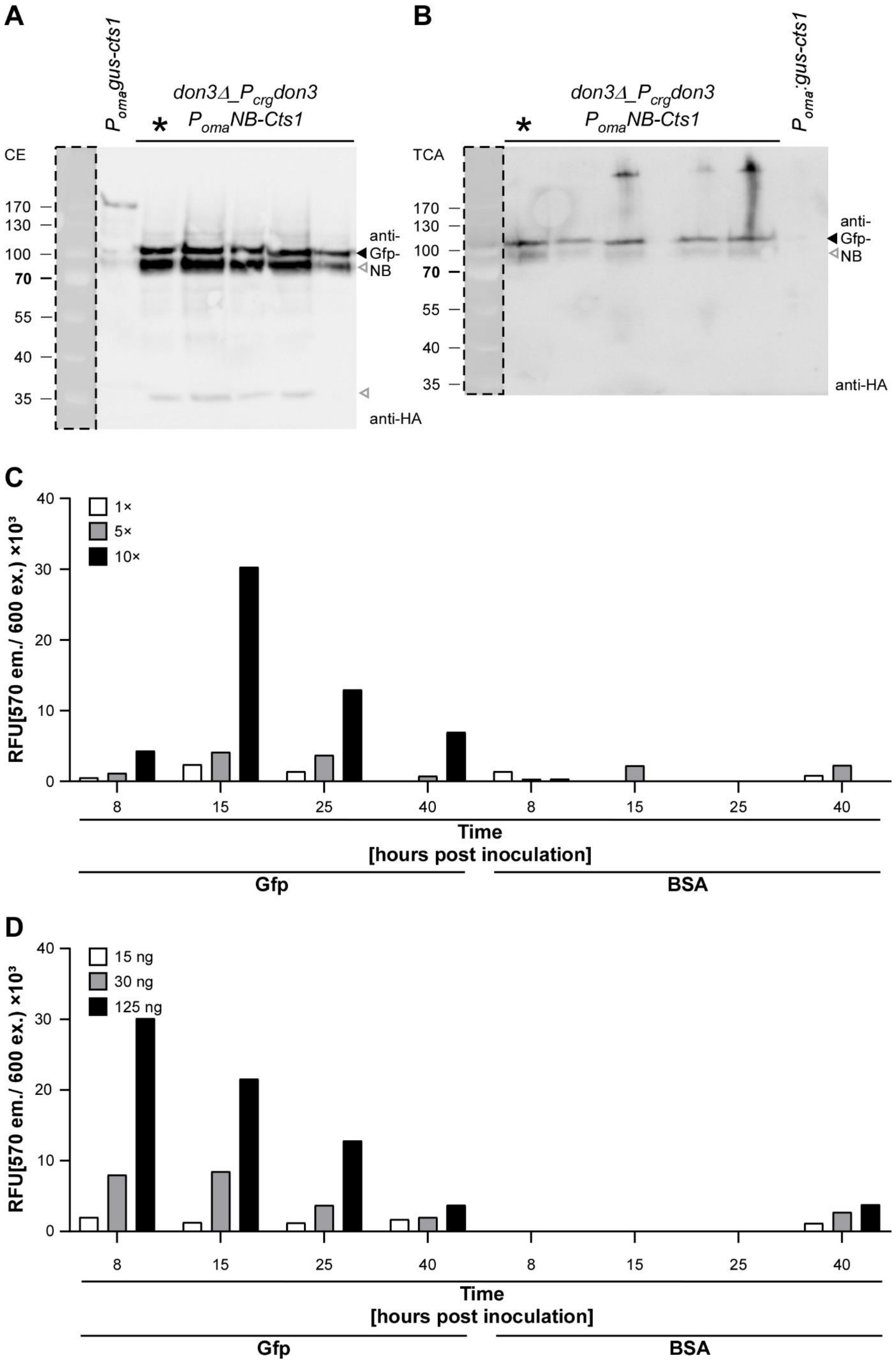
A
B
P_{oma}gus-cts1
don3Δ_P_{crg}don3
P_{oma}NB-Cts1
*
CE
TCA
170
130
100
70
55
40
35
anti-
Gfp-
NB
anti-HA
P_{oma}:gus-cts1
C
D
RFU[570 em./ 600 ex.) ×10³
40
30
20
10
0
1×
5×
10×
15 ng
30 ng
125 ng
8
15
25
40
Time
[hours post inoculation]
Gfp
BSA

Figure S7. Additional data on unconventional secretion of anti-Gfp nanobodies. (**A**,**B**) Western blot analysis of different anti-Gfp nanobody secreting strains. (**A**) Cell extracts (CE) and (**B**) TCA precipitated supernatants (TCA) of AB33don3Δ/ P_{crg}don3/NB-Cts1 candidates are shown. Cells were cultivated in medium containing arabinose. Primary an-tibodies against HA were used to detect the respective proteins (anti-HA). Black arrowheads indicate NB-Cts1 signal (pre-dicted size: 75 kDa), open gray arrowheads indicate known degradation products [28]. AB33 Gus-Cts1 was used as a positive control. Proteins run slightly higher than expected. This phenomenon was observed before [14]. Dashed area indicates digitization of protein marker with 70% opacity. Asterisks indicate selected candidate for further experiments. (**C**,**D**) Complete result of ELISA experiment which is shown in Figure 7C and D. Evaluation of autoinduction process using anti-Gfp nanobody fused to Cts1 (NB-Cts1) as read-out. Strain AB33don3Δ/P_{crg}don3/NB-Cts1 was cultivated in CM medium supplemented with 1% glucose and 1% arabinose, buffered with 0.1 M MES. The inoculated culture was split into 5 individual flasks for harvest of cell extracts, supernatant and online growth monitoring in a BioLector. Supernatant and cell pellets were collected at defined time points, unconventionally secreted NB-Cts1 was IMAC purified from supernatant and cell extracts were prepared. For purified supernatant and cell extracts ELISA was performed using purified Gfp as antigen and bovine serum albumin (BSA) as a negative control to monitor unspecific retention of NB-Cts1 in wells. (**A**) ELISA of purified supernatants. 1×, 5× and 10× concentrated purified supernatants. (**B**) ELISA of cell extracts containing defined protein amounts of 15 ng, 30 ng and 125 ng whole cell extract.

References

14. Stock, J.; Sarkari, P.; Kreibich, S.; Brefort, T.; Feldbrügge, M.; Schipper, K. Applying unconventional secretion of the endochitinase Cts1 to export heterologous proteins in *Ustilago maydis*. *J. Biotechnol.* **2012**, *161*, 80–91, doi:10.1016/j.jbiotec.2012.03.004.
28. Terfrüchte, M.; Reindl, M.; Jankowski, S.; Sarkari, P.; Feldbrügge, M.; Schipper, K. Applying unconventional secretion in *Ustilago maydis* for the export of functional nanobodies. *Int. J. Mol. Sci.* **2017**, *18*, 937, doi:10.3390/ijms18050937.
30. Reindl, M.; Stock, J.; Hussnaetter, K.P.; Genc, A.; Brachmann, A.; Schipper, K. A novel factor essential for unconventional secretion of chitinase Cts1. *Front. Microbiol.* **2020**, *11*, 1529, doi:10.1101/2020.02.07.938613.
